# Supplementary material for: Anaemia among adolescent girls, pregnant and lactating women in the southern rural region of Bangladesh: Prevalence and risk factors
Source: PLoS One. 2024 Jul 10;19(7):e0306183. doi: 10.1371/journal.pone.0306183 (PMC11236138; doi:10.1371/journal.pone.0306183)
Supplement: S1 File — (PDF) [file pone.0306183.s001.pdf]

Assessment of anaemia status among pregnant and lactating women and adolescent girls in southern Bangladesh

Nutrition Research Division, icddr,b

Respondent ID (Mother/adolescent girls): ☐☐☐-☐☐☐☐

**DEMOGRAPHIC & SOCIO-ECONOMIC STATUS QUESTION FORM**	
Fieldworker ID	☐☐
Date of interview (DD/MM/YY)	☐☐ ☐☐ ☐☐
Name of District	
Name of Upazila	
Name of Union	
Name of village	
Village code	☐☐
Type of respondent<br>Pregnant women=01<br>Lactating Women=02<br>Adolescent girls=03	☐☐

**Module D1: DEMOGRAPHIC QUESTIONS**			
#	Question	Code	**Response**
Questions for household head<br>(Here the household head means the person who stays with the family and gives day to day decisions.)			
D1.1	What is your relationship to …………? [participant's name]<br><br>***(If response 06=Self, Skip to question D1.9)***	01= Father<br>02= Mother<br>03= Grandmother<br>04= Grandfather<br>05= Sibling<br>06= Self<br>07= Offspring<br>08= Spouse<br>09= In law<br>10= Others	☐☐
D1.1.a	If other, please specify-		
D1.2	What is your age?	In years	☐☐
D1.3	Sex	01=Male 02=Female	☐☐

D1.4	Household head's occupation	00= Not currently working<br>01= Land owner<br>02= Agriculture worker<br>03= Fisherman<br>04= Home based manufacturer<br>05= Rickshaw/Van driver<br>06= Car/Bus/Truck/Cng driver<br>07= Construction worker<br>08= Factory/Garments worker<br>09= Day labourer<br>10= Business<br>11= Others	☐☐
D1.4.a.	If other, please specify		
D1.5	What is your marital Status?<br>*If never married, skip to question 7.*	01=Never married<br>02= Married<br>03=Divorced<br>04=Widowed	☐☐
D1.6	How old were you when you got married for the first time?	In years	☐☐
D1.7	Have you ever attended any educational institutes? *If no, skip to question 11.*	01=Yes 02=No	☐☐
D1.8	If yes, how many years of education have you completed?		☐☐
Questions D1.9- D1.15 are for the pregnant/lactating mother/adolescent girls. If the mother is temporarily unavailable, return to the household at another time to complete these questions.			
D1.9	What is your age?	In years	☐☐
D1.10	Mother's occupation.	00= Not currently working<br>01= Housewife<br>02= Garments worker<br>03= Beggar<br>04= Home based manufacturer<br>05= House help<br>06= Brick breaking<br>07= Business<br>08= Others	☐☐
D1.10.a	If other, please specify		
D1.11	What is your relation with household head?	01= Father<br>02= Mother<br>03= Grandmother<br>04= Grandfather<br>05= Sibling<br>06= Self<br>07= Offspring<br>08= Spouse<br>09= In law<br>10= Others	☐☐

D1.11a	If other, please specify		
D1.12	What is your marital Status? *If never married, skip to question D1.14.*	01= Never married<br>02= Married<br>03=Divorced<br>04=Widowed	
D1.13	How old were you when you get married for the first time?	In years	
D1.14	Have you ever attended any educational institutes? *If no, skip to question D1.16.*	01=Yes 00=No	
D1.15	If yes, how many years of education have you completed?	In years	
Questions D1.16- D1.20 should be addressed to the participant's husband. Complete these questions only if the household head and participant's husband is not the same person. If both are same person, skip to question D2.1.			
D1.16	What is your relationship to …………………………? *[participant's name]*	01= Father<br>02= Mother<br>03= Grandmother<br>04= Grandfather<br>05= Sibling<br>06= Self<br>07= Offspring<br>08= Spouse<br>09= In law<br>10= Others	
D1.16.a	If other, please specify-		
D1.17	What is your age?	In years	
D1.18	What is your marital Status? *If never married, skip to question D1.31.*	01= Never married<br>02= Married<br>03= Divorced<br>04=Widowed	
D1.19	Have you ever attended any educational institutes? *If no, skip to question D1.36.*	01=Yes 02=No	
D1.20	If yes, how many years of education have you completed?	In years	
**Module D2: SOCIO-ECONOMIC STATUS QUESTIONS**			
D2.1	How long is your family living in this house?	Years: Months	
D2.2	How many rooms are there in your house?	01-15 (Rooms)	
D2.3	How many rooms in this household are used for sleeping?	01-10 (Rooms)	
D2.4	How many people usually sleep in this household?	01-30 (People)	

D2.5	Does this household have a separate space to use as a kitchen?	Yes = 01; No = 00	
D2.6	If yes, where is the kitchen situated?	01= In a separate room within the house<br>02= In veranda<br>03= In courtyard<br>04= In separate house<br>05= Others	
D2.7	If other, please specify-		
D2.8	What type of cooking stove do you/your family mainly use?	01= Kerosene stove<br>02= Gas stove<br>03= Open fire<br>04= Open fire or stove with chimney or hood<br>05= Closed stove with chimney<br>06= Electric heaters<br>07= Other	
D2.9	If other, please specify		
D2.10	Do you/your household pay any domestic workers?	Yes = 01; No = 00	
D2.11	What is the main source of drinking water for members of your household?	01= Piped into dwelling<br>02= Piped to yard/plot<br>03= Public tap/stand pipe<br>04= Tube well or borehole<br>05= Protected well<br>06= Unprotected well<br>07= Surface water (river/ dam/ lake/pond/ stream/canal/irrigation canal)<br>08=Other	
D2.12	If other, please specify		
D2.13	What is the main source of water used by your household for other purposes such as cooking and hand-washing?	01= Piped into dwelling<br>02= Piped to yard/plot<br>03= Public tap/stand pipe<br>04= Tube well or borehole<br>05= Protected well<br>06= Unprotected well<br>07= Surface water (river/ dam/ lake/pond/ stream/canal/irrigation canal)<br>08=Other	
D2.14	If other, please specify		
D2.15	Do you/your family pay or barter separately for water?	01=Yes 00=No	

D2.16	Do you treat your water in any way to make it safer to drink?<br>*If no skip to question D2.19.*	01=Yes 00=No	
D2.17	If yes, what do you usually do to the water to make it safer to drink?	01=Let it stand and settle<br>02=Solar disinfection<br>03=Use water filter (ceramic /sand/ composite/etc.)<br>04=Strain through a cloth<br>05=Add bleach/chlorine 06=Boil<br>07=Other	
D2.18	If other, please specify-		
D2.19	Do you wash your hands with soap after helping your child defecate?<br><br>(In case of the adult participant, if she/he is not a mother/father of such child, please write NA)	01=Never<br>02=Rarely<br>03=Sometimes<br>04=Always	
D2.20	Do you wash your hands with soap before preparing food?	01=Never<br>02=Rarely<br>03=Sometimes<br>04=Always	
D2.21	Do you wash your hands with soap after using the toilet?	01=Never<br>02=Rarely<br>03=Sometimes<br>04=Always	
D2.21.a	Do you have access to a handwashing device in/near the latrine?	01=Yes 00=No	
D2.21.b	Do you have access to a handwashing device at the dining area	01=Yes 00=No	
D2.22	Do you use toilet paper?	01=Never<br>02=Rarely<br>03=Sometimes<br>04=Always	
D2.23	What kind of toilet facility do members of your household usually use?	01= No facility/bush/field or bucket toilet 02= Pit latrine without flush (Without slab)<br>03= Flush to piped sewer system<br>04= Flush to septic tank<br>05= Flush to pit latrine<br>06= Flush to somewhere else<br>07= Pit latrine without slab<br>08= Other	

D2.23.a	If other, please specify-		
D2.24	Do you share this toilet facility with other households? *If no, skip to question D2.26.*	01=Yes 00=No	
D2.25	If yes, how many households use this toilet facility?		
Now I am going to ask you about whether your household owns a series of items. Please respond yes if you own the item and it is in working form. If you do not own the item or own it but it is broken or not working, please respond no.			
D2.26	Does your household have an iron (either charcoal or electric)?	01=Yes 00=No	
D2.27	Does your household have a mattress?	01=Yes 00=No	
D2.28	Does your household have a chair or bench?	01=Yes 00=No	
D2.29	Does your household have a sofa?	01=Yes 00=No	
D2.30	Does your household have a table?	01=Yes 00=No	
D2.31	Does your household have an electric fan?	01=Yes 00=No	
D2.32	Does your household have a radio or transistor?	01=Yes 00=No	
D2.33	Does your household have a computer?	01=Yes 00=No	
D2.34	Does your household have a television?	01=Yes 00=No	
D2.35	Does your household have a mobile telephone?	01=Yes 00=No	
D2.36	Does your household have a refrigerator?	01=Yes 00=No	
D2.37	Does your household have a motor cycle?	01=Yes 00=No	
D2.38	Does any member of your household have a bank account (savings or current)?	01=Yes 00=No	
D2.39	Does any member this household own any agricultural land? *If no, D2.42.*	01=yes 00=No	

D2.40	If yes, do you get any financial help from this land?	01=Yes 00=No	
D2.41	How much agricultural land does this household own?		
D2.42	Does your household own chickens or ducks?	01=Yes 00=No	
D2.43	Main material of the floor (observation)	01= Earth/sand/clay/mud/dung<br>02= Wood<br>03= Ceramic tiles<br>04= Cement/Concrete<br>05= Other	
D2.43.a	If other, please specify-		
D2.44	Main material of the roof (observation)	01= No roof 05= Concrete<br>00= Thatch 06= Tiles<br>03= Metal 07= Slate<br>04= Wood 08= Other	
D2.44.a	If other, please specify-		
D2.45	Main material of the exterior walls (observation; if multiple material is present write the costly one)	01=No walls 05= Bamboo<br>00=Mud 06=Metal<br>03=Wood 07=Other<br>04=Cement/concrete	
D2.45.a	If other, please specify-		
D2.46	What is the average monthly income for the entire household (in taka)?<br>*Start writing from right		

Women Questionnaire: Module E			
**Characteristics of the respondent**			
**Module E1: Background of the respondent**			
I would like to ask you some questions about you, your husband, your current pregnancy/ last pregnancy			
Respondents must be pregnant and lactating women (Adolescents only ask questions from ***E1.1 - E1.7****)*			
Q#	Questions/Instructions	Coding categories	Codes
E1.1	Do you read newspapers or magazines?	01=Yes 00=No (if no, skip to E1.7)	

E1.1.1	If yes. how often do you read a newspapers or magazines	01 = Almost every day<br>02 = At least once a week<br>03 = Less than once a week	[ ][ ]
E1.2	Do you listen to the radio?	1= Yes, 0=No (if no, skip to E1.8)	[ ][ ]
E1.2.1	If yes, how often do you listen to radio?	01 = Almost every day<br>02 = At least once a week<br>03 = Less than once a week	[ ][ ]
E1.3	Do you watch television?	01= Yes, 00=No (if no, skip to E1.9)	[ ][ ]
E1.3.1	If yes, how often do you watch television?	01 = Almost every day<br>02 = At least once a	[ ][ ]
E1.4	What is your religion?	01 = Muslim<br>02 = Hindu<br>03 = Buddhist<br>04 = Christian	[ ][ ]
E1.5	Are you currently working? (Payment in cash or kind)	01= Yes, 00=No (If, no, skip to section E2)	[ ][ ]
E1. 6	What kind of work do you mainly do?<br>Verbatim:______________________<br>(Record what the mother says then categories from the list)	01 = Professional<br>02 = Domestic servant<br>03 = Technical<br>04 = Business<br>05 = Factory worker<br>06 = Semi-skilled labour/service<br>0 7= Unskilled labour<br>08 = Poultry/ cattle raising<br>09 = Home-based manufacturing	[ ][ ]
E1.7	How much do you earn each month?	Taka:	Tk.[__][__][__][__][__]
**Module E2:** Current pregnancy history of the respondent (please ask this section only to pregnant women)			
Section E2: Current pregnancy		Please check the screening form if any discrepancies observed contact your supervisor.	
Now I would like to ask you about your current pregnancy			
E2.1	How many months pregnant are you?	Number of months:	[ ][ ]

**Module E3:** Current history of pregnancy mother (Please ask this section only before delivery)			
E3.1	Did you visit anyone for pregnancy related medical checkup?	01= Yes, 00=No	
E3.2	If yes, whom did you visit<br>Multiple answer can be possible<br>A. Qualified doctor<br>B. Nurse/Midwife/ Paramedic<br>C. Medical Assistant (MA) /Sub-Assistant<br>Community Medical Officer (SACMO)<br>D. Trained TBA<br>E. Untrained TBA (Dai)<br>F. Unqualified Doctor	01= Yes, 00=No	
E3.3	Where did you mainly receive antenatal care during your pregnancy?	<u>HOME</u><br>01 = Own home<br>02= Other home<br><u>PUBLIC SECTOR</u><br>03 = Hospital or medical college hospital<br>04 = Community clinic<br>05 = Satellite clinic/ EPI outreach<br><u>NGO SECTOR</u><br>06 = NGO static clinic<br>07 = NGO satellite clinic<br><u>PRIVATE SECTOR</u><br>08 = Private hospital<br>09 = Maternity clinic<br>10 = Private clinic<br>11= Qualified doctor<br>10 = Traditional doctor<br>13 = Kobiraj<br>14 = Pharmacy	
E3.4	How many times did you receive ANC when you were pregnant with (NAME)?	Number of times:<br><br>Don't know ….98	
E3.5	How many months pregnant were you when you first received antenatal care (ANC)?	Months:<br><br>Don't know …98	
**Module E4 is applicable for lactating women**			
E4.1	Where did you mainly receive post-natal check up after your delivery?	<u>HOME</u><br>01 = Own home<br>02= Other home<br><u>PUBLIC SECTOR</u>	

		03 = Hospital or medical college hospital<br>04 = Community clinic<br>05 = Satellite clinic/ EPI outreach<br>NGO SECTOR<br>06 = NGO static clinic<br>07 = NGO satellite clinic<br>PRIVATE SECTOR<br>08 = Private hospital<br>09 = Maternity clinic<br>10 = Private clinic<br>11= Qualified doctor<br>10 = Traditional doctor<br>13 = Kobiraj<br>14 = Pharmacy	
E4.2	How many times did receive post-natal checkup after your delivery?	Number of times:<br><br>Don't know ….98	[ ][ ]
**Module E5: Micronutrient supplementation (This section is applicable for pregnant and lactating women)**			
**Q. No.**	**Questions/Instructions**	**Coding categories**	**Code/Skip**
E5.1	Current physical state of the mother?	01= Pregnant,<br>02= Lactating (0-6 months) (If 2, skip to E7.1)<br>03= Lactating (6 -24 months) (If 3, skip to E7.5)	[ ][ ]
E5.2	During your last pregnancy did you take any IFA/ iron syrup (Show the sample)?	01= Yes, 00=No	[ ][ ]
E5.2.1	If yes, how frequent are you taking IFA// syrup?	01= Daily<br>02= One or more a week<br>03= One or more months<br>98= Don't know	[ ][ ]
E5.3	Estimated number of iron tablet intake	Number: [__|__]	

**WOMEN DIETARY DIVERSITY - MODULE F**<br>*Which of the following foods did you eat yesterday?*			
**Q. No.**	**Questions/Instructions**	**Coding Categories**	**Code**
F1.1	Foods made from grains *(e.g., rice, bread, wheat, barley, corn, pasta/noodles, porridge, sorghum, millet, couscous)*	1= yes, 0 = No	[__]

F1.2	White roots and tubers or plantains *(e.g. White potatoes, white yams, manioc/cassava/yucca, cocoyam, taro roots or tubers, plantains*)	1= yes, 0 = No	[__]
F1.3	Pulses *(e.g. Beans, peas, lentils, hummus, tofu, tempeh*)	1= yes, 0 = No	[__]
F1.4	Nuts and seeds *(e.g. Groundnut/peanut, cashew, walnut, Baobab seeds, chia seeds, flaxseed*)	1= yes, 0 = No	[__]
F1.5	Milk and milk products *(e.g. Milk, cheese or yoghurt*)	1= yes, 0 = No	[__]
F1.6	Organ meats *(e.g. Liver, kidney, heart or other organ meats or blood-based foods)*	1= yes, 0 = No	[__]
F1.7	Flesh meats *(e.g. goat/ beef; chicken/poultry)*	1= yes, 0 = No	[__]
F1.8	Fish and seafood *(e.g. Fresh, frozen or dried fish, shrimp, clams)*	1= yes, 0 = No	[__]
F1.9	Eggs *(e.g. Eggs from poultry or any other bird)*	1= yes, 0 = No	[__]
F1.10	Dark green leafy vegetable *(e.g. Kale, mustard greens, spinach, amaranth greens, chicory, broccoli, Swiss chard*	1= yes, 0 = No	[__]
F1.11	Vitamin A rich vegetables or tubers *(e.g.* Pumpkin, carrots, squash or sweet potatoes)	1= yes, 0 = No	[__]
F1.12	Other vegetables (e.g., Beets, cabbage, cauliflower, celery, cucumbers, eggplant, zucchini, radish, tomato, mushroom)	1= yes, 0 = No	[__]
F1.13	Vitamin A rich fruits *(e.g.* Ripe mango, ripe papaya)	1= yes, 0 = No	[__]
F1.14	Other fruits (e.g., Apple, avocado, banana, baobab fruit, berries, pineapple, orange, watermelon, berries, guava, coconut flesh, tangerine)	1= yes, 0 = No	[__]
F1.15	Sweet foods (e.g., Chocolates, candies, pastries, cakes, biscuits, cookies, ice cream)	1= yes, 0 = No	[__]
F1.16	Sweet beverages (e.g., Soft drinks, sodas, fruit juices, chocolate drinks, energy drinks, Sweetened tea, sweetened coffee, or sweetened herbal drinks)		
F1.17	Oils/fats *(e.g. cooking fat or oil, butter, ghee, margarine)?*	1= yes, 0 = No	[__]
F1.18	Condiments and seasonings (e.g., Onion, parsley, garlic, herbs, spices, bouillon cubes, tomato paste)	1= yes, 0 = No	[__]

ANTHROPOMETRY- MODULE (G)

Interviewer: measure the height and weight and record in appropriate box. (only for adolescent girls)		
**Q. No.**	**Indicators**	**Measurement**
G1.1	Height in cm (1st measurement)	[ ][ ][ ] [ ]
G1.2	Height in cm (2nd measurement)	[ ][ ][ ] [ ]
G 2.1	Weight in Kg (1st measurement)	[ ][ ] [ ] [ ]
G 2.2	Weight in Kg (2md measurement)	[ ][ ] [ ] [ ]

Haemoglobin: MODULE K

**No**	**Hemoglobin**	**Unit**	**Measurement**
		Gram/DL	

Assessment of anaemia status among pregnant and lactating women and adolescent girls in southern Bangladesh

Nutrition Research Division, icddr, b

উত্তরদাতা আইডি (মা/কিশোরী মেয়ে): ☐☐☐-☐☐☐☐

**DEMOGRAPHIC & SOCIO-ECONOMIC STATUS QUESTION FORM**	
মাঠকর্মী আইডি	☐☐
সাক্ষাৎকারের তারিখ (DD/MM/YY)	☐☐ ☐☐ ☐☐
জেলার নাম	
উপজেলার নাম	
ইউনিয়নের নাম	
গ্রামের নাম	
গ্রামের কোড	☐☐
উত্তরদাতার ধরণ<br><br>গর্ভবতী মহিলা = 01<br>স্তন্যদানকারী মহিলা = 02<br>কিশোরী বালিকা = 03	☐☐

**Module D1: DEMOGRAPHIC QUESTIONS**			
**#**	**প্রশ্ন**	**কোড**	**উত্তর**
**পরিবারের প্রধানের জন্য প্রশ্ন**<br>**(এখানে পরিবারের প্রধান মানে সেই ব্যক্তি যিনি পরিবারের সাথে থাকেন এবং প্রতিদিনের সিদ্ধান্ত দেন)**			
D1.1	আপনার সাথে [অংশগ্রহণকারীর নাম] এর সম্পর্ক কি?<br><br>*(যদি উত্তর স্বয়ং হয়, তাহলে D1.9 নম্বর প্রশ্ন এ যান)*	০১= পিতা<br>০২= মা<br>০৩= দাদি<br>০৪= দাদা<br>০৫= ভাইবোন<br>০৬= স্বয়ং<br>০৭= সন্তান<br>০৮= পতি/ পত্নী<br>০৯= বৈবাহিক সূত্রে আত্মীয়<br>১০= অন্যান্য	☐☐
D1.1. a	যদি অন্যান্য হয়, তাহলে উল্লেখ করুন-		

D1.2	আপনার বয়স কত?	(বছরে লিখো)	☐☐
D1.3	লিঙ্গ	০১= পুরুষ<br>০২= মহিলা	☐☐
D1.4	গৃহকর্তার পেশা	০০= বর্তমানে কাজ করছে না<br>০১= জমির মালিক<br>০২= কৃষি কর্মী<br>০৩= জেলে<br>০৪= বাসা ভিত্তিক প্রস্তুতকারক<br>০৫= রিকশা/ভ্যান চালক<br>০৬= গাড়ি/বাস/ট্রাক/সিএনজি ড্রাইভার<br>০৭= নির্মাণ শ্রমিক<br>০৮= কারখানা/গার্মেন্টস শ্রমিক<br>০৯= দিনমজুর<br>১০= ব্যবসা<br>১১= অন্যান্য	☐☐
D1.4.a.	যদি অন্যান্য হয়, তাহলে উল্লেখ করুন-		
D1.5	আপনার বৈবাহিক অবস্থা কি?<br>*(যদি এখনো বিয়ে না করে থাকেন, তাহলে* D1.7 *নম্বর প্রশ্ন তে চলে যান)*	০১= কখনো বিয়ে করেনি<br>০২= বিবাহিত<br>০৩= তালাকপ্রাপ্ত<br>০৪= বিধবা	☐☐
D1.6	আপনি যখন প্রথম বিয়ে করেছিলেন তখন আপনার বয়স কত ছিল?	(বছরে লিখো)	☐☐
D1.7	আপনি কি কখনো কোন শিক্ষা প্রতিষ্ঠানে গিয়েছেন?<br>*(যদি না হয়, তাহলে* D1.11 *নম্বর প্রশ্ন তে চলে যান)*	০১= হ্যাঁ<br>০২= না	☐☐
D1.8	যদি হ্যাঁ হয়, তাহলে আপনি সর্বোচ্চ কোন ক্লাস পাস করেছেন?	(বছরে লিখো)	☐☐
প্রশ্ন D1.9- D1.15 গর্ভবতী/ স্তন্যদানকারী মা/ কিশোরী মেয়েদের জন্য। যদি মা সাময়িকভাবে অনুপস্থিত থাকে, তাহলে এই প্রশ্নগুলি সম্পূর্ণ করার জন্য অন্য সময়ে পরিবারের কাছে ফিরে আসুন			
D1.9	আপনার বয়স কত?	(বছরে লিখো)	☐☐
D1.10	পেশা	০০= বর্তমানে কাজ করছে না<br>০১= গৃহিণী<br>০২= গার্মেন্টস কর্মী<br>০৩= ভিক্ষুক<br>০৪= বাসা ভিত্তিক প্রস্তুতকারক<br>০৫= বাড়ির কাজে সাহায্য<br>০৬= ইট ভাঙ্গা<br>০৭= ব্যবসা<br>০৮= অন্যান্য	☐☐
D1.10.a	যদি অন্যান্য হয়, তাহলে উল্লেখ করুন-		

D1.11	পরিবারের প্রধানের সাথে আপনার সম্পর্ক কি?	০১= পিতা<br>০২= মা<br>০৩= দাদি<br>০৪= দাদা<br>০৫= ভাইবোন<br>০৬= স্বয়ং<br>০৭= সন্তান<br>০৮= পতি/ পত্নী<br>০৯= বৈবাহিক সূত্রে আত্মীয়<br>১০= অন্যান্য	
D1.11a	যদি অন্যান্য হয়, তাহলে উল্লেখ করুন-		
D1.12	আপনার বৈবাহিক অবস্থা কি?<br><br>*(যদি কখনো বিয়ে না করে থাকেন, তাহলে D1.14 নম্বর প্রশ্ন তে চলে যান)*	০১= কখনো বিয়ে করেনি<br>০২= বিবাহিত<br>০৩= তালাকপ্রাপ্ত<br>০৪= বিধবা	
D1.13	আপনি যখন প্রথমবার বিয়ে করেছিলেন তখন আপনার বয়স কত ছিল?	(বছরে লিখো)	
D1.14	আপনি কি কখনো কোন শিক্ষা প্রতিষ্ঠানে গিয়েছেন?<br>*(যদি না হয়, তাহলে D1.16 নম্বর প্রশ্ন তে চলে যান)*	০১= হ্যাঁ<br>০২= না	
D1.15	যদি হ্যাঁ হয়, তাহলে আপনি সর্বোচ্চ কোন ক্লাস পাস করেছেন?	(বছরে লিখো)	
প্রশ্ন D1.16- D1.20 অংশগ্রহণকারীর স্বামীকে সম্বোধন করতে হবে। পরিবারের প্রধান এবং অংশগ্রহণকারীর স্বামী একই মানুষ না হলেই শুধুমাত্র এই প্রশ্নগুলি সম্পূর্ণ করুন। যদি উভয়ই একই ব্যক্তি হয়, প্রশ্ন D2.1 তে চলে যান			
D1.16	আপনার সাথে [অংশগ্রহণকারীর নাম] এর সম্পর্ক কি?	০১= পিতা<br>০২= মা<br>০৩= দাদি<br>০৪= দাদা<br>০৫= ভাইবোন<br>০৬= স্বয়ং<br>০৭= সন্তান<br>০৮= পতি/ পত্নী<br>০৯= বৈবাহিক সূত্রে আত্মীয়<br>১০= অন্যান্য	
D1.16.a	যদি অন্যান্য হয়, তাহলে উল্লেখ করুন-		
D1.17	আপনার বয়স কত?	(বছরে লিখো)	
D1.18	আপনার বৈবাহিক অবস্থা কি?<br><br>*(যদি এখনো বিয়ে না করে থাকেন, তাহলে D1.19 নম্বর প্রশ্ন তে চলে যান)*	০১= কখনো বিয়ে করেনি<br>০২= বিবাহিত<br>০৩= তালাকপ্রাপ্ত<br>০৪= বিপত্নীক	

D1.19	আপনি কি কখনো কোন শিক্ষা প্রতিষ্ঠানে গিয়েছেন?<br>*(যদি না হয়, তাহলে D2.1প্রশ্ন তে চলে যান)*	০১= হ্যাঁ<br>০২= না	
D1.20	যদি হ্যাঁ হয়, তাহলে আপনি সর্বোচ্চ কোন ক্লাস পাস করেছেন?	(বছরে লিখো)	
**Module D2: SOCIO-ECONOMIC STATUS QUESTIONS**			
D2.1	আপনার পরিবার কতদিন এই বাড়িতে বসবাস করছে?	বছরঃ মাস	
D2.2	আপনার বাড়িতে কয়টি কক্ষ আছে?	০১-১৫ (রুম)	
D2.3	এই পরিবারের কয়টি কক্ষ ঘুমানোর জন্য ব্যবহৃত হয়?	০১-১০ (রুম)	
D2.4	এই বাড়িতে সাধারণত কতজন মানুষ ঘুমায়?	০১-৩০ (মানুষ)	
D2.5	এই পরিবারের কি রান্নাঘর হিসেবে ব্যবহারের জন্য আলাদা জায়গা আছে?	০১= হ্যাঁ<br>০০= না	
D2.6	যদি হ্যাঁ হয়, রান্নাঘর কোথায় অবস্থিত?	০১= ঘরের মধ্যে একটি আলাদা ঘরে<br>০২= বারান্দায়<br>০৩= উঠানে<br>০৪= আলাদা বাড়িতে<br>০৫= অন্যান্য	
D2.7	যদি অন্যান্য হয়, তাহলে উল্লেখ করুন-		
D2.8	আপনি/আপনার পরিবার প্রধানত কোন ধরনের রান্নার চুলা ব্যবহার করেন?	০১= কেরোসিনের চুলা<br>০২= গ্যাস চুলা<br>০৩= খোলা আগুন<br>০৪= খোলা আগুন / চিমনি বা ফণা দিয়ে চুলা<br>০৫= চিমনি দিয়ে বন্ধ চুলা<br>০৬= বৈদ্যুতিক উনান<br>০৭= অন্যান্য	
D2.9	যদি অন্যান্য হয়, তাহলে উল্লেখ করুন-		
D2.10	আপনি/আপনার পরিবার কি কোন গৃহকর্মীকে বেতন দেন?	০১= হ্যাঁ<br>০০= না	
D2.11	আপনার পরিবারের সদস্যদের জন্য পানীয় জলের প্রধান উৎস কি?	০১= বাসস্থান এ পাইপ ব্যবহার করা (ম্যাক্স টপওয়াটার)<br>০২= ম্যাক্স ওভারহেড ট্যাংক<br>০৩= আঙ্গিনা / প্লটে পাইপ করা<br>০৪= পাবলিক ট্যাপ/স্ট্যান্ড পাইপ<br>০৫= নলকূপ বা বোরহোল<br>০৬= সুরক্ষিত কুয়া<br>০৭= অরক্ষিত কুয়া<br>০৮= পৃষ্ঠের জল (নদী/ বাঁধ/ হ্রদ/ পুকুর/ স্রোত/ খাল/ সেচ খাল)<br>০৯= অন্যান্য	

D2.12	যদি অন্যান্য হয়, তাহলে উল্লেখ করুন-		
D2.13	আপনার পরিবারের অন্যান্য কাজে যেমন রান্না এবং হাত ধোয়ার জন্য ব্যবহৃত পানির প্রধান উৎস কী?	০১= বাসস্থান এ পাইপ ব্যবহার করা (ম্যাক্স ট্যাপওয়াটার)<br>০২= ম্যাক্স ওভারহেড ট্যাংক<br>০৩= আঙ্গিনা / প্লটে পাইপ করা<br>০৪= পাবলিক ট্যাপ/স্ট্যান্ড পাইপ<br>০৫= নলকূপ বা বোরহোল<br>০৬= সুরক্ষিত কুয়া<br>০৭= অরক্ষিত কুয়া<br>০৮= পৃষ্ঠের জল (নদী/ বাঁধ/ হ্রদ/ পুকুর/ স্রোত/ খাল/ সেচ খাল)<br>০৯= অন্যান্য	☐☐
D2.14	যদি অন্যান্য হয়, তাহলে উল্লেখ করুন-		
D2.15	আপনি/আপনার পরিবার কি পানির জন্য আলাদাভাবে অর্থ প্রদান বা বিনিময় করেন?	০১= হ্যাঁ<br>০০= না	☐☐
D2.16	আপনি কি পানিকে নিরাপদ করার জন্য কোন উপায়ে পানি বিশুদ্ধ করেন?<br><br>(*যদি না হয়, তাহলে D2.19 নম্বর প্রশ্ন তে চলে যান*)	০১= হ্যাঁ<br>০০= না	☐☐
D2.17	যদি হ্যাঁ হয়, পানিকে নিরাপদ করার জন্য আপনি সাধারণত কী করেন?	০১= স্থির হতে দেয়া<br>০২= সৌর জীবাণুমুক্তকরণ<br>০৩= জল ফিল্টার ব্যবহার করে (সিরামিক/বালি/যৌগিক/ইত্যাদি)<br>০৪= কাপড় দিয়ে ছেকে<br>০৫= ব্লিচ/ক্লোরিন ব্যবহার<br>০৬= ফুটান<br>০৭= অন্যান্য	☐☐
D2.18	যদি অন্যান্য হয়, তাহলে উল্লেখ করুন-		
D2.19	আপনার সন্তানকে মলত্যাগে সাহায্য করার পর আপনি কি সাবান দিয়ে হাত ধোবেন?<br><br>(*প্রাপ্তবয়স্ক অংশগ্রহণকারীর ক্ষেত্রে, যদি সে/ সে এই ধরনের সন্তানের মা/ বাবা না হয়, তাহলে অনুগ্রহ করে “প্রযোজ্য নয়” লিখুন*)	০১= কখনোই না<br>০২= কদাচিৎ<br>০৩= মাঝে মাঝে<br>০৪= সর্বদা	☐☐
D2.20	আপনি কি খাবার প্রস্তুত করার আগে সাবান দিয়ে হাত ধৌত করেন?	০১= কখনোই না<br>০২= কদাচিৎ<br>০৩= মাঝে মাঝে<br>০৪= সর্বদা	☐☐
D2.21	আপনি কি টয়লেট ব্যবহারের পর সাবান দিয়ে হাত ধৌত করেন?	০১= কখনোই না<br>০২= কদাচিৎ<br>০৩= মাঝে মাঝে<br>০৪= সর্বদা	☐☐

D2.21.a	আপনার ল্যাট্রিনের কাছে কি হাত ধোয়ার কোনো উৎস আছে?	০১= হ্যাঁ<br>০০= না	
D2.21.b	আপনার কি ডাইনিং এর কাছে হাত ধোয়ার কোনো উৎস আছে?	০১= হ্যাঁ<br>০০= না	
D2.21.c	যদি হ্যাঁ হয়, এটা কি ম্যাক্সি-বেসিন?	০১= হ্যাঁ<br>০০= না	
D2.21.c	যদি হ্যাঁ হয়, আপনি কি আমাকে বলতে পারেন যে, হাত ধোয়ার ওই উৎস স্থাপনের জন্য কত টাকা খরচ হয়েছিল?	টাকা ___/___/___/___/	
D2.22	আপনি কি টয়লেট পেপার ব্যবহার করেন?	০১= কখনোই না<br>০২= কদাচিৎ<br>০৩= মাঝে মাঝে<br>০৪= সর্বদা	
D2.23	আপনার পরিবারের সদস্যরা সাধারণত কোন ধরনের টয়লেট সুবিধা ব্যবহার করেন?	০১= কোন সুবিধা নেই / ঝোপ / মাঠ / বালতি টয়লেট<br>০২= ফ্লাশ ছাড়াই পিট ল্যাট্রিন (স্ল্যাব ছাড়া)<br>০৩= পাইপযুক্ত নর্দমা ব্যবস্থায় ফ্লাশ<br>০৪= সেপটিক ট্যাঙ্কে ফ্লাশ<br>০৫= পিট ল্যাট্রিনে ফ্লাশ<br>০৬= অন্য কোথাও ফ্লাশ<br>০৭= স্ল্যাব ছাড়াই পিট ল্যাট্রিন<br>০৮= অন্যান্য	
D2.23.a	যদি অন্যান্য হয়, তাহলে উল্লেখ করুন-		
D2.24	আপনি কি অন্যান্য পরিবারের সাথে এই টয়লেট সুবিধা শেয়ার করেন?<br>*(যদি না হয়, D2.26 নম্বর প্রশ্ন এ যান)*	০১= হ্যাঁ<br>০০= না	
D2.25	যদি হ্যাঁ হয়, কয়টি পরিবার এই টয়লেট সুবিধা ব্যবহার করে?		
**এখন আমি আপনাকে জিজ্ঞাসা করতে যাচ্ছি যে আপনার পরিবারের নীচে উল্লিখিত এই ধরণের জিনিসের মালিক কিনা। দয়া করে 'হ্যাঁ' উত্তর দিন, যদি আপনি জিনিসটির মালিক হন এবং এটি কার্যকরী আকারে থাকে। যদি আপনি জিনিসটির মালিক না হন বা এটির মালিক না হন তবে এটি ভেঙে গেছে বা কাজ করছে না, দয়া করে 'না' উত্তর দিন**			
D2.26	আপনার বাড়িতে কি স্ত্রী আছে (চারকোল বা বৈদ্যুতিক)?	০১= হ্যাঁ<br>০০= না	
D2.27	আপনার পরিবারের কারো কি কোনো গদি আছে?	০১= হ্যাঁ<br>০০= না	
D2.28	আপনার পরিবারে কি চেয়ার বা বেঞ্চ আছে?	০১= হ্যাঁ<br>০০= না	
D2.29	আপনার পরিবারে কি কোনো সোফা আছে?	০১= হ্যাঁ<br>০০= না	

D2.30	আপনার পরিবারে কি কোনো টেবিল আছে?	০১= হ্যাঁ<br>০০= না	
D2.31	আপনার বাড়িতে কি কোনো বৈদ্যুতিক পাখা আছে?	০১= হ্যাঁ<br>০০= না	
D2.32	আপনার পরিবারে কি রেডিও বা ট্রানজিস্টর আছে?	০১= হ্যাঁ<br>০০= না	
D2.33	আপনার পরিবারে কি কোনো কম্পিউটার আছে?	০১= হ্যাঁ<br>০০= না	
D2.34	আপনার বাড়িতে কি কোনো টেলিভিশন আছে?	০১= হ্যাঁ<br>০০= না	
D2.35	আপনার পরিবারের কারো কি কোনো মোবাইল / টেলিফোন আছে?	০১= হ্যাঁ<br>০০= না	
D2.36	আপনার বাড়িতে কি কোনো ফ্রিজ আছে?	০১= হ্যাঁ<br>০০= না	
D2.37	আপনার পরিবারে কি কারো কোনো মোটরসাইকেল আছে?	০১= হ্যাঁ<br>০০= না	
D2.38	আপনার পরিবারের কোন সদস্যের কি ব্যাংক অ্যাকাউন্ট আছে (সঞ্চয় বা বর্তমান)?	০১= হ্যাঁ<br>০০= না	
D2.39	এই পরিবারের কোন সদস্য কি কোন কৃষি জমির মালিক?<br>*(যদি না হয়, তাহলে D2.42 নম্বর প্রশ্ন) তে চলে যান)*	০১= হ্যাঁ<br>০০= না	
D2.40	যদি হ্যাঁ হয়, আপনি কি এই জমি থেকে কোন আর্থিক সাহায্য পান?	০১= হ্যাঁ<br>০০= না	
D2.41	এই পরিবারের কত টা কৃষিজমি আছে?		
D2.42	আপনার পরিবারের কেও কি মুরগি বা হাঁসের মালিক?	০১= হ্যাঁ<br>০০= না	
D2.43	মেঝের প্রধান উপাদান (পর্যবেক্ষণ)	০১= পৃথিবী/বালি/মাটি/কাদা/গোবর<br>০২= কাঠ<br>০৩= সিরামিক টাইলস<br>০৪= সিমেন্ট/কংক্রিট	
D2.43.a	যদি অন্যান্য হয়, তাহলে উল্লেখ করুন-		
D2.44	ছাদের প্রধান উপাদান (পর্যবেক্ষণ)	০১= দেয়াল নেই ০৫= কংক্রিট<br>০২= তালপাতা ০৬= টাইলস<br>০৩= ধাতু ০৭= সেলেট পাথর<br>০৪= কাঠ ০৮= অন্যান্য	
D2.44.a	যদি অন্যান্য হয়, তাহলে উল্লেখ করুন-		

D2.45	বাইরের দেয়ালের প্রধান উপাদান (পর্যবেক্ষণ; যদি একাধিক উপকরণ উপস্থিত থাকে, তবে ব্যয়বহুল উপকরণ টি লিখুন)	০১= দেয়াল নেই<br>০২= কাদা<br>০৩= কাঠ<br>০৪= সিমেন্ট/কংক্রিট<br>০৫= বাঁশ<br>০৬= ধাতু<br>০৭= অন্যান্য	☐☐
D2.45.a	যদি অন্যান্য হয়, তাহলে উল্লেখ করুন-		
D2.46	পুরো পরিবারের জন্য গড় মাসিক আয় (টাকায়) কত?<br>*ডান দিক থেকে লেখা শুরু করুন	☐☐☐☐☐☐☐☐	

Women Questionnaire: Module E

Characteristics of the respondent

Module E1: Background of the respondent

আমি আপনাকে, আপনার স্বামী, আপনার বর্তমান গর্ভাবস্থা/ শেষ গর্ভাবস্থা সম্পর্কে কিছু প্রশ্ন করতে চাই

*উত্তরদাতা গর্ভবতী এবং স্তন্যদানকারী নারী হতে হবে (কিশোরীদের জন্য শুধুমাত্র **E1.1** - E1.7 থেকে প্রশ্ন করুন)*

Q#	প্রশ্ন/নির্দেশাবলী	কোডিং বিভাগ	কোড
E1.1	আপনি কি সংবাদপত্র বা পত্রিকা পড়তে পারেন?<br>*(যদি না হয় তাহলে E 1.7 নম্বর প্রশ্ন) তে চলে যান)*	০১= হ্যাঁ<br>০০= না	☐☐
E1.1.1	যদি হ্যাঁ হয় তাহলে আপনি সাধারণত কখন সংবাদপত্র বা পত্রিকা পড়েন?	০১ = প্রায় প্রতিদিন<br>০২ = সপ্তাহে অন্তত একবার<br>০৩ = সপ্তাহে একবারের কম	☐☐
E1.2	আপনি কি রেডিও শুনেন?<br>*(যদি না হয়, তাহলেউ ১.৮ নম্বর প্রশ্ন) তে চলে যান)*	০১= হ্যাঁ<br>০০= না	☐☐
E1.2.1	যদি হ্যাঁ হয় তাহলে আপনি কখন কখন রেডিও শুনেন?	০১ = প্রায় প্রতিদিন<br>০২ = সপ্তাহে অন্তত একবার<br>০৩ = সপ্তাহে একবারের কম	☐☐
E1.3	আপনি কি টেলিভিশন দেখেন?<br>*(যদি না হয়, তাহলেউ ১.৯ নম্বর প্রশ্ন) তে চলে যান)*	০১= হ্যাঁ<br>০০= না	☐☐
E1.3.1	যদি হ্যাঁ হয় তাহলে আপনি কখন কখন টেলিভিশন দেখেন?	০১ = প্রায় প্রতিদিন<br>০২ = সপ্তাহে অন্তত একবার<br>০৩ = সপ্তাহে একবারের কম	☐☐
E1.4	আপনার ধর্ম কি?	০১ = মুসলিম<br>০২ = হিন্দু<br>০৩ = বৌদ্ধ<br>০৪ = খ্রিস্টান	☐☐

E1.5	আপনি কি এখন কাজ করছেন? (নগদ বা প্রকারে অর্থ প্রদান)<br><br>*(যদি না হয়, তাহলেউ ২ নম্বর প্রশ্ন) তে চলে যান)*	০১= হ্যাঁ<br>০০= না	[ ][ ]
E1. 6	আপনি প্রধানত কোন ধরনের কাজ করেন?<br>শব্দগত: ___________________________<br>(মা যা বলেন তা রেকর্ড করুন তারপর তালিকা থেকে বিভাগগুলি)	০১ = পেশাদার<br>০২ = গৃহকর্মী<br>০৩ = প্রযুক্তিগত<br>০৪ = ব্যবসা<br>০৫ = কারখানার শ্রমিক<br>০৬ = অর্ধশিক্ষিত শ্রমিক / সেবা<br>০৭ = অদক্ষ শ্রমিক<br>০৮ = হাঁস/ গবাদি পশু পালন<br>০৯ = বাসা ভিত্তিক উত্পাদন	[ ][ ]
E1.7	আপনি প্রতি মাসে কত উপার্জন করেন?	টাকা	[__][__][__][__][__]
**Module E2:** **উত্তরদাতার বর্তমান গর্ভাবস্থার ইতিহাস (অনুগ্রহ করে এই অংশটি শুধুমাত্র গর্ভবতী মহিলাদের জিজ্ঞাসা করুন)**			
Section E2: **বর্তমান গর্ভাবস্থা**		যদি কোন অসঙ্গতি পরিলক্ষিত হয় তাহলে আপনার সুপারভাইজারের সাথে যোগাযোগ করুন।	
**এখন আমি আপনার বর্তমান গর্ভাবস্থা সম্পর্কে জিজ্ঞাসা করতে চাই**			
E2.1	আপনি কত মাস ধরে গর্ভবতী	মাসের সংখ্যা	[ ][ ]
**Module E3:** গর্ভবতী মায়ের বর্তমান ইতিহাস (অনুগ্রহ করে শুধুমাত্র প্রসবের আগে এই নিয়ে জিজ্ঞাসা করুন)			
E3.1	আপনি কি গর্ভাবস্থার মেডিকেল চেক আপের জন্য কারো সাথে পরামর্শ করেছেন বা দেখাইছেন?	০১= হ্যাঁ<br>০০= না	[ ][ ]
E3.2	যদি হ্যাঁ, আপনি কার কাছে গিয়েছিলেন<br>একাধিক উত্তর দেওয়া সম্ভব<br>A. যোগ্য ডাক্তার<br>B. নার্স/ মিডওয়াইফ/ প্যারামেডিক<br>C. মেডিকেল সহকারী (এমএ) /উপ-সহকারী কমিউনিটি মেডিকেল অফিসার (SACMO)<br>D. প্রশিক্ষিত TBA<br>E. প্রশিক্ষণহীন TBA (Dai)<br>F. অযোগ্য ডাক্তার	০১= হ্যাঁ ০০= না<br>০১= হ্যাঁ ০০= না<br>০১= হ্যাঁ ০০= না<br>০১= হ্যাঁ ০০= না<br>০১= হ্যাঁ ০০= না<br>০১= হ্যাঁ ০০= না	[ ][ ]<br>[ ][ ]<br>[ ][ ]<br>[ ][ ]<br>[ ][ ]<br>[ ][ ]

E3.3	আপনি আপনার গর্ভাবস্থায় প্রধানত প্রসবকালীন যত্ন কোথায় পেয়েছিলেন?	**বাড়ি**<br>১ = নিজের বাড়ি<br>২ = অন্য বাড়ি<br>**সরকারি খাত**<br>৩ = হাসপাতাল বা মেডিকেল কলেজ হাসপাতাল<br>৪ = কমিউনিটি ক্লিনিক<br>৫ = স্যাটেলাইট ক্লিনিক/ ইপিআই আউটরিচ<br>**এনজিও সেক্টর**<br>৬ = এনজিও স্ট্যাটিক ক্লিনিক<br>৭ = এনজিও স্যাটেলাইট ক্লিনিক<br>**ব্যক্তিগত খাত**<br>৮ = বেসরকারি হাসপাতাল<br>৯ = প্রসূতি ক্লিনিক<br>১০ = প্রাইভেট ক্লিনিক<br>১১ = যোগ্য ডাক্তার<br>১০ = গতানুগতিক ডাক্তার<br>১৩ = কবিরাজ<br>১৪ = ফার্মেসী	☐☐
E3.4	আপনি যখন (নাম) গর্ভবতী ছিলেন তখন আপনি কতবার প্রসবকালীন যত্ন পেয়েছিলেন?	সংখ্যা<br>জানি না... ৯৮	☐☐
E3.5	আপনি যখন প্রথম প্রসবকালীন যত্ন পেয়েছিলেন তখন আপনি কত মাসের গর্ভবতী ছিলেন?	মাস:<br>জানি না... ৯৮	☐☐
**Module E4: এটি স্তন্যদানকারী মহিলাদের জন্য প্রযোজ্য**			
E4.1	প্রসবের পর আপনি প্রসব-পরবর্তী চেক-আপ প্রধানত কোথায় পেয়েছেন?	**বাড়ি**<br>১ = নিজের বাড়ি<br>২ = অন্য বাড়ি<br>**সরকারি খাত**<br>৩ = হাসপাতাল বা মেডিকেল কলেজ হাসপাতাল<br>৪ = কমিউনিটি ক্লিনিক<br>৫ = স্যাটেলাইট ক্লিনিক/ ইপিআই আউটরিচ<br>**এনজিও সেক্টর**<br>৬ = এনজিও স্ট্যাটিক ক্লিনিক<br>৭ = এনজিও স্যাটেলাইট ক্লিনিক<br>**ব্যক্তিগত খাত**<br>৮ = বেসরকারি হাসপাতাল<br>৯ = প্রসূতি ক্লিনিক<br>১০ = প্রাইভেট ক্লিনিক<br>১১ = যোগ্য ডাক্তার<br>১০ = গতানুগতিক ডাক্তার<br>১৩ = কবিরাজ<br>১৪ = ফার্মেসী	☐☐
E4.2	আপনার প্রসবের পর কতবার প্রসব-পরবর্তী চেকআপ পেয়েছেন?	সংখ্যা<br>জানি না... ৯৮	☐☐

Module E5: Micronutrient supplementation<br>(এই বিভাগটি গর্ভবতী এবং স্তন্যদানকারী মহিলাদের জন্য প্রযোজ্য)			
**Q. No.**	**Questions/Instructions**	**Coding categories**	**Code/Skip**
E5.1	মায়ের বর্তমান শারীরিক অবস্থা?	০১= গর্ভবতী<br>০২= স্তন্যদানকারী মা (০-৬ মাস)<br>০৩= স্তন্যদানকারী মা (৭-২৪ মাস)	[ ][ ]
E5.2	গর্ভাবস্থায় আপনি কোন ফলিক এসিড ট্যাবলেট/ আয়রন সিরাপ আয়রন গ্রহণ করছেন?<br>(আপনি আমাকে নমুনা দেখাতে পারেন)	০১= হ্যাঁ<br>০০= না	[ ][ ]
E5.2.1	যদি হ্যাঁ, আপনি কত ঘন ঘন আয়রন, ফলিক এসিড ট্যাবলেট গ্রহণ করছেন?	০১= দৈনিক<br>০২= সপ্তাহে একদিন বা এর অধিক<br>০৩= এক মাস বা এর অধিক<br>৯৮= জানি না	[ ][ ]
E5.2	আয়রন ট্যাবলেট খাওয়ার আনুমানিক সংখ্যা	সংখ্যা: [__|__]	

WOMEN DIETARY DIVERSITY - MODULE F<br>*গতকাল আপনি নিম্নলিখিত খাবারগুলো থেকে কোনগুলো খেয়েছেন-*			
F1.1	শস্য থেকে তৈরি খাবার (যেমন, চাল, রুটি, গম, বার্লি, ভুট্টা, পাস্তা/নুডুলস, পোরিজ, খোসাযুক্ত শস্যদানা, চালের খুত)	1= হ্যাঁ, 0 = না	[__]
F1.2	সাদা মূল বা কন্দ জাতীয় খাবার (যেমন সাদা আলু, ম্যানিওক/কাসাভা/ইউক্কা, মুলা, কচু)	1= হ্যাঁ, 0 = না	[__]
F1.3	ডাল জাতীয় খাবার (যেমন শুকনা কলাই বা বিচি, শুকনো মটরশুটি বা শিম, মসুর ডাল, সবুজ ছোলা, ঘাস মটর, সয়াবিন, এগুলোর বীজ এবং এগুলো দিয়ে তৈরি খাবার)	1= হ্যাঁ, 0 = না	[__]
F1.4	যেকোনো বাদাম বা বাদাম জাতীয় খাবার (যেমন চিনাবাদাম, কাজু বাদাম, পেস্তা বাদাম, আখরোট, চিয়াসিড, ফ্ল্যাক্সসিড)	1= হ্যাঁ, 0 = না	[__]
F1.5	যে কোন দুধ বা দুগ্ধজাত দ্রব্য (যেমন দুধ, পনির, দই বা অন্যান্য দুধের দ্রব্য (কিন্তু মাখন, আইসক্রিম, ক্রিম বা টক ক্রিম নয়)	1= হ্যাঁ, 0 = না	[__]
F1.6	প্রাণীর অঙ্গ থেকে তৈরি যেকোনো মাংস (যেমন কলিজা (লিভার), গুরদা, মগজ, ফুস্‌ফুস, কিডনি, হার্ট বা অন্যান্য অঙ্গের মাংস বা রক্ত-ভিত্তিক খাবার)	1= হ্যাঁ, 0 = না	[__]
F1.7	অন্য কোনো ধরনের মাংস (যেমন গরু, মহিষ, ছাগল, ভেড়া কোয়েল, কবুতর, হাঁস, মুরগি, ও অন্যান্য পাখির মাংস)	1= হ্যাঁ, 0 = না	[__]
F1.8	যেকোনো মাছ বা সামুদ্রিক খাবার, তা তাজা হোক বা শুকনো (যেমন তাজা মাছ, শুটকি মাছ, শামুক, ঝিনুক, রুই, কাতলা, কার্প, কৈ, তেলাপিয়া, টেংড়া, চাপিলা, চিংড়ি, মাছের ডিম)	1= হ্যাঁ, 0 = না	[__]
F1.9	যেকোনো ডিম (যেমন হাঁস, মুরগি বা অন্য কোনো পাখির ডিম)	1= হ্যাঁ, 0 = না	[__]
F1.10	যেকোনো গাঢ় সবুজ শাক (যেমন পালং শাক, লাল শাক, কচু শাক, ডাটা শাক, পুঁই শাক, লাউ শাক, কলমি শাক, পাট শাক, সজনে পাতা, লেটুস পাতা)	1= হ্যাঁ, 0 = না	[__]
F1.11	ভিটামিন এ সমৃদ্ধ সবজি ও কন্দ (যেমন কুমড়া, গাজর, কমলা, স্কোয়াশ বা মিষ্টি আলু যেগুলির ভিতরে হলুদ বা কমলা)	1= হ্যাঁ, 0 = না	[__]
F1.12	অন্যান্য সবজি (যেমন পেঁয়াজ, টমেটো, বেগুন, চালকুমড়া, লাউ, পটল, করলা, ঝিংগা, চিচিঙ্গা, বরবটি, শশা, কাকরোল, শিম, ঢেড়শ, ফুলকপি, ডাটা)	1= হ্যাঁ, 0 = না	[__]
F1.13	স্থানীয়ভাবে পাওয়া যায় এমন ভিটামিন-এ সমৃদ্ধ ফল (যেমন পাকা আম, পাকা তাল, জাম, পাকা পেপে, এপ্রিকট, বেল, জাম্বুরা, ডেওয়া, গাব, তরমুজ ইত্যাদি যে কোন ফল ভিতরে গাঢ় হলুদ বা কমলা)	1= হ্যাঁ, 0 = না	[__]

F1.14	অন্যান্য ফল (যেমন পেয়ারা, কাঠাল, লিচু, কলা, আনাওরস, খেজুর, কামরাঙ্গা)	1= হ্যাঁ, 0 = না	[__]
F1.15	যে কোনো মিষ্টি জাতীয় খাবার (যেমন চকোলেট, ক্যান্ডি, পেস্ট্রি, কেক, বিস্কুট, কুকিজ, আইসক্রিম)	1= হ্যাঁ, 0 = না	[__]
F1.16	মিষ্টি পানীয় (যেমন, কোমল পানীয়, সোডা, ফলের রস, চকোলেট পানীয়, শক্তি পানীয়, মিষ্টি চা, মিষ্টি কফি, বা মিষ্টি ভেষজ পানীয়)	1= হ্যাঁ, 0 = না	
F1.17	তেল / চর্বি (যেমন রান্নার তেল, মাখন, ঘি, ডালডা)	1= হ্যাঁ, 0 = না	[__]
F1.18	মসলা (যেমন, পেঁয়াজ, পার্সলে, রসুন, ভেষজ, মশলা, বোউলন কিউব, টমেটো পেস্ট)	1= হ্যাঁ, 0 = না	[__]

ANTHROPOMETRY- MODULE (G)

Interviewer: measure the height and weight and record in appropriate box. (only for adolescent girls)

Q. No.	Indicators	একক	পরিমাপ
G1.1	উচ্চতা (সেমি) (১ম পরিমাপ)	সেমি	[ ][ ][ ] [ ]
G1.2	উচ্চতা (সেমি) (২য় পরিমাপ)	সেমি	[ ][ ][ ] [ ]
G 2.1	ওজন (কেজি) (১ম পরিমাপ)	কেজি	[ ][ ][ ] [ ]
G 2.2	ওজন (কেজি) (১ম পরিমাপ)	কেজি	[ ][ ][ ] [ ]

Haemoglobin: MODULE K

**No**	**হিমোগ্লোবিন**	**একক**	**পরিমাপ**
		Gram/DL	[ ][ ][ ]
